# Social context mediates the expression of a personality trait in a gregarious lizard

**DOI:** 10.1007/s00442-022-05269-7

**Published:** 2022-09-29

**Authors:** Jack A. Brand, Annalise C. Naimo, Marcus Michelangeli, Jake M. Martin, Andrew Sih, Bob B. M. Wong, David G. Chapple

**Affiliations:** 1 School of Biological Sciences, Monash University, Melbourne, VIC Australia; 2 Department of Environmental Science and Policy, University of California, Davis, CA USA; 3 Department of Wildlife, Fish, and Environmental Studies, Swedish University of Agricultural Sciences, Umeå, Sweden

**Keywords:** Among-individual variation, Behavioural syndrome, Behavioural type, Individual plasticity, Within-individual variation

## Abstract

The social environment is a key factor that influences behavioural traits across a wide array of species. Yet, when investigating individual differences in behaviour, studies tend to measure animals in isolation from other conspecifics—even in social species. Surprisingly, whether behavioural traits measured in isolation are predictive of individual-level behaviour when in social groups is still poorly understood. Here, we repeatedly measured risk-taking behaviour (i.e. boldness; 741 total trials) in both the presence and absence of conspecifics in a social lizard, the delicate skink (*Lampropholis delicata*). Further, we manipulated food availability during group trials to test whether the effect of the social environment on risk-taking behaviour was mediated by competition over resources. Using 105 lizards collected from three independent populations, we found that individual risk-taking behaviour was repeatable when measured in either social isolation or within groups both with and without food resources available. However, lizards that were bolder during individual trials were not also bolder when in groups, regardless of resource availability. This was largely driven by individual differences in social behavioural plasticity, whereby individual skinks responded differently to the presence of conspecifics. Together, this resulted in a rank order change of individual behavioural types across the social conditions. Our results highlight the importance of the social environment in mediating animal personality traits across varying levels of resource availability. Further, these findings suggest that behavioural traits when measured in isolation, may not reflect individual variation in behaviour when measured in more ecologically realistic social groups.

**Supplementary Information:**

The online version contains supplementary material available at 10.1007/s00442-022-05269-7.

## Introduction

It is widely accepted that individuals within populations consistently differ from one another in their average-level behaviour (Dingemanse and Reale 2005; Wolf and Weissing 2012). For example, some individuals tend to be more prone to risk-taking or are more active and exploratory than other conspecifics in the population (e.g. Jolles et al. 2019; Michelangeli et al. 2019; Brand et al. 2021a, b). These ‘personality traits’ or ‘behavioural types’ are typically heritable (Dingemanse et al. 2002; Dochtermann et al. 2015), can affect organismal fitness (Moirón et al. 2019; Munson et al. 2020), as well as species interactions and biological invasions (Chapple et al. 2012; Sih et al. 2012; Wolf and Weissing 2012). Thus, consistent individual differences in behaviour can have important implications for ecology and evolution.

However, while behavioural types often demonstrate some level of consistency through time and across contexts (Sih et al. 2004; Payne et al. 2021), research has highlighted that individuals can also consistently differ in their behavioural plasticity in response to changing environmental conditions (Cornwell et al. 2019; Mitchell and Biro 2017; Dingemanse et al. 2012). The social environment is one example of an ecologically relevant context that may play an important role in mediating individual behaviour (Webster and Ward 2011; Montiglio et al. 2017; Rudin et al. 2019; Zhang et al. 2020; Mason et al. 2021; Niemelä and Santostefano 2015). For example, previous research in zebrafish (*Danio rerio*) reported that exploratory behaviour was modified by the presence of a social partner (Guayasamin et al. 2017). In particular, the authors found that zebrafish adjusted their behaviour to match that of their partner’s during paired trials (Guayasamin et al. 2017). Similar findings were also reported in Gouldian finches (*Erythrura gouldiae*) where birds matched the risk-taking behaviour of their partner when in social pairs (King et al. 2015). Such studies highlight the importance of the social environment in altering key behavioural traits.

Interestingly, recent work has suggested that the presence of conspecifics may also mediate both among- and within-individual behavioural variation. Research in southern field crickets (*Gryllus bimaculatus*), for example, found that animals housed socially displayed higher levels of behavioural repeatability in their aggression than conspecifics housed in social isolation (Jäger et al. 2019). This higher behavioural repeatability in social environments was driven by both an increase in variation among individuals and a decrease in within-individual behavioural variability (Jäger et al. 2019). In contrast, while three-spined sticklebacks (*Gasterosteus aculeatus*) held in social isolation were significantly repeatable in their risk-taking behaviour, this was not the case for fish housed within social groups (Jolles et al. 2016). While the effect of social experience on behaviour may differ among species, these findings suggest that interaction with conspecifics may play a key role in shaping individual behavioural types.

Despite this, most studies investigating behavioural types typically measure animals in isolation from other conspecifics (Webster and Ward 2011; Martin and McCallum 2021). This is true even for social species. A key question, therefore, is whether individual behavioural types expressed in social isolation (i.e. in standardized behavioural assays) are truly reflective of their behavioural type in more ecologically realistic social settings (Niemelä and Dingemanse 2014). This is important, as the effect of social context on individual behaviour may have vital implications for organismal fitness. For example, differences in behavioural types can mediate survival in wild populations (Moirón et al. 2019). Individual variation in social behavioural plasticity (i.e. when individuals are not consistent across social contexts) may, therefore, result in differential survival across different social conditions. Indeed, prior research in wild three-spined sticklebacks found that the influence of risk-taking behavioural types on survival was dependent upon an individual’s social niche (Pearish et al. 2019). More specifically, in a mark-recapture experiment, Pearish et al. (2019) found that bold fish were more likely to survive in the wild when in shoals, whereas shyer fish displayed increased survival when alone. Taken together, this research suggests that social conditions may mediate the fitness consequences of behavioural types. However, despite the importance of the social environment in influencing the fitness consequences of behaviour, whether individuals are consistent in their behavioural types across changing social conditions is still not well known.

Moreover, the effects of the social environment on behaviour may be further influenced by resource availability. For example, three-spined sticklebacks were slower moving and formed less cohesive shoals in the presence of food versus the absence of food (Jolles et al. 2018). Similarly, social conflict and aggressive interactions are more likely to be observed in larger social groups due to higher within-group competition for available resources (e.g. Kaspersson et al. 2010). Thus, resource availability likely plays an important role in determining the effect of the social context on individual behavioural types. However, very few studies have repeatedly tested the behaviour of individuals across both social and foraging conditions—key ecological contexts critical to species survival—to determine whether individual behavioural types are consistent across these ecologically relevant environmental conditions.

Accordingly, we investigated the consistency of individual behavioural types across changing social and foraging conditions in the delicate skink (*Lampropholis delicata*). Delicate skinks are a small, gregarious lizard species that can be found at high population densities (Cogger 2014). Moreover, the species is frequently encountered within loosely formed conspecific groups (Downes and Hoefer 2004; Michelangeli et al. 2017; Littlewood et al. 2021) and often lay their eggs within large communal nests (Chapple et al. 2014). Previous research has found a strong effect of group size in modulating the antipredator behaviour of delicate skinks, suggesting that social conditions may mediate risk-taking behaviour in this species (Downes and Hoefer 2004). However, whether individuals are consistent in their risk-taking behaviour across these changing social conditions is not well known. Therefore, we repeatedly tested individuals across different social contexts to determine whether individual risk-taking behaviour, when measured in isolation, predicts risk-taking when in social groups. We also tested individuals within groups across changing foraging conditions to determine whether the effect of the social environment on behaviour is mediated by resource availability. Based on previous research in other taxa (e.g. Magnhagen and Bunnefeld 2009; Jolles et al. 2017), we predicted that individuals which were bolder when measured in isolation would also be bolder when measured within social groups, both in the presence and absence of food. Further, we predicted that individual risk-taking behaviour would be consistent within groups across the foraging conditions.

## Methods

### Study species and animal husbandry

Delicate skinks are native to eastern Australia but have successfully established multiple invasive populations throughout the Pacific (Hawaiian Islands, New Zealand, Lord Howe Island). We collected adult male skinks with intact and/or fully regenerated tails from three populations located within or on the fringe of state/national parks across the species invasive Hawaiian range (Hawai’i [19°26 N, 155°13 W]: *n* = 36; Kaua’i [22°07 N, 159°39 W]: *n* = 36; O’ahu [21°18 N, 157°49 W]: *n* = 35; total: *n* = 107). Skinks were observed in densities of up to 20 individuals per m^2^ in their invasive range (A.C. Naimo, pers. obs), suggesting that they frequently encounter large numbers of conspecifics in the wild. Lizards were caught using a combination of mealworm fishing and hand capture, methods previously shown not to bias samples towards particular behavioural types (Michelangeli et al. 2016b). Following capture, we determined the sex of each lizard via eversion of the hemipenes. Lizards were marked with a unique identification code using Visual Implant Elastomers (Northwest Marine Technology, Shaw Island, WA, U.S.A.) and measured for snout-vent length (SVL; snout to the cloaca; range: 32–44 mm) using digital callipers. We only tested males as the behaviour of female *Lampropholis* skinks is highly sensitive to their reproductive state (Shine 2003) and we only tested individuals with intact and/or fully regenerated tails to control for previously reported effects of tail loss on behaviour (Michelangeli et al. 2020).

Following capture, skinks were transported to the University of California, Davis, where they were housed in communal groups within a controlled-temperature room (22.5 ± 0.5 °C) for at least 1 week prior to the start of behavioural trials. Housing enclosures (40 × 30 × 37 cm) contained newspaper and plastic pots for shelter. Similarly, each enclosure was provided with UV lighting between 08:00 and 18:00 h, while a terracotta tile that was heated (22–32 °C) with heat-tape between 08:00 and 17:00 h allowed skinks to behaviourally thermoregulate. Each lizard was supplied with three small (~ 1 cm) crickets (*Acheta domesticus*) dusted in a vitamin supplement (Reptivite™), three times a week, while water was available ad libitum.

### Behavioural experiments

#### Individual trials

**Fig. 1:**
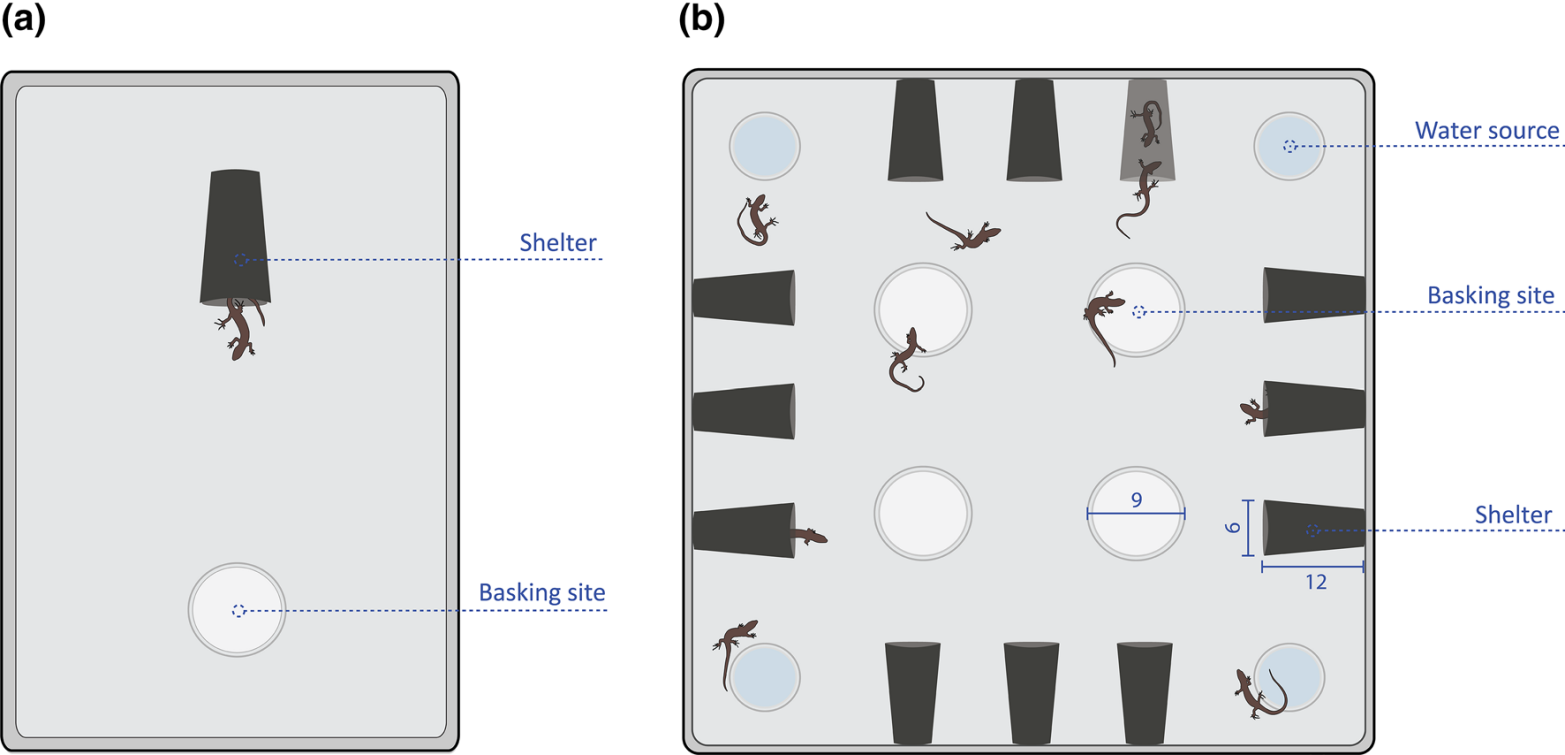
Schematic diagram of both **a** individual and **b** group behavioural assays for delicate skinks (*Lampropholis delicata*). During individual trials we recorded the time taken to emerge from the shelter as a measure of risk-taking behaviour. Similarly, risk-taking behaviour was measured as the total time spent sheltering during group trials. All measurements are in cm. Lizards not to scale. Figure was adapted from Brand et al. (2021a, b)

All lizards were individually tested for risk-taking behaviour following previously established protocols for *Lampropholis* skinks (Michelangeli et al. 2019; Michelangeli et al. 2016a, b). Briefly, experimental arenas (55 × 35 × 25 cm) contained a basking site at one end and a shelter at the other (Fig. 1a). Lizards were initially introduced into the centre of the arena and allowed to acclimate in a clear container for 10 min. After acclimation, an experimental observer would gently chase each lizard with a rod until it entered the shelter. This was done to ensure that each skink started the trial from within the shelter. Over the following 60 min trial, we recorded the time taken to emerge from the shelter, which has been previously used as a measure of risk-taking behaviour in a wide variety of species (Michelangeli et al. 2019; Myles-Gonzalez et al. 2015; Royauté et al. 2020). Individuals that did not emerge from the shelter during the trial were given a score of 3600 s (i.e. total duration of the assay; occurred in 9.5% of observations). Experimental arenas were washed and wiped clean with an odourless, non-toxic detergent between trials to avoid the accumulation of conspecific cues. Assays were repeated after 4 days to measure short-term behavioural repeatability.

#### Group trials

Following individual trials, lizards were divided into groups of 12 individuals (except one group of 11) all from the same population with three groups per population. This group size was chosen as it is within the observed population densities found in the species’ Hawaiian range (i.e. up to 20 skinks per m^2^; A.C. Naimo pers. obs), and has been used in previous studies investigating how group size mediates delicate skink antipredator behaviour (Downes and Hoefer 2004). Group trials were conducted over 3 days in large (100 × 100 × 30 cm) arenas fitted with 12 shelter sites (12 × 6 cm), four basking sites (9 cm diameter; maintained at 29.5 °C ± 0.5 °C), and four water sources (Fig. 1b). Before trials, individuals were marked with unique colour combinations of non-toxic paint to track their identity within the group. Lizards were allowed to acclimate as a group in a clear container (25 × 12 cm) located in the centre of the arena for 10 min before they were released and allowed to freely explore. During individual trials, we measured re-emergence latencies from a shelter as this is a standard measure of risk-taking behaviour in *Lampropholis* skinks (Michelangeli et al. 2019; Naimo et al. 2021). However, in the group trials, it was too disruptive and infeasible to chase all 12 skinks each into one of the 12 separate shelters at the same time to run a parallel standardized assay for emergence times. Similarly, if we had, instead, quantified skink emergence latencies from different shelters (in some cases, shared with other interacting skinks) at different times after the experiment began, this would also have added several potential confounds to the assessment of consistent individual differences in boldness. We, therefore, measured the total time that each individual spent sheltering over the duration of the 20 min trial as a more complete measure of risk-taking behaviour during group trials. Total sheltering time is another widely used measure of boldness (Polverino et al. 2021; Ortiz-Jimenez et al. 2022), including in reptiles (Stahlschmidt et al. 2016; Skinner and Miller 2020).

After this initial 20 min trial, lizards were kept in the arenas for 4 h, after which 12 small (1 cm) crickets were introduced into the centre of the arena. Total sheltering time was scored again for 20 min. This process was repeated each day for 3 days and allowed us to measure the behaviour of each lizard in both the presence and absence of food resources. All behavioural trials were video-recorded (JVC Everio GZ-E100) for later analysis using the event-logging software BORIS (Friard and Gamba 2016). Videos were scored blind to individual identity. While lizards in the current experiment were previously used in a study comparing population differences in skink activity rates when in groups (i.e. no individual trials), none of the data presented here were previously reported in Brand et al. (2021a). Instead, we chose to focus on shelter use across varying social conditions in the present study as we were interested in the consistency of risk-taking behaviour when measured during individual and group assays.

### Statistical analysis

**Fig. 2:**
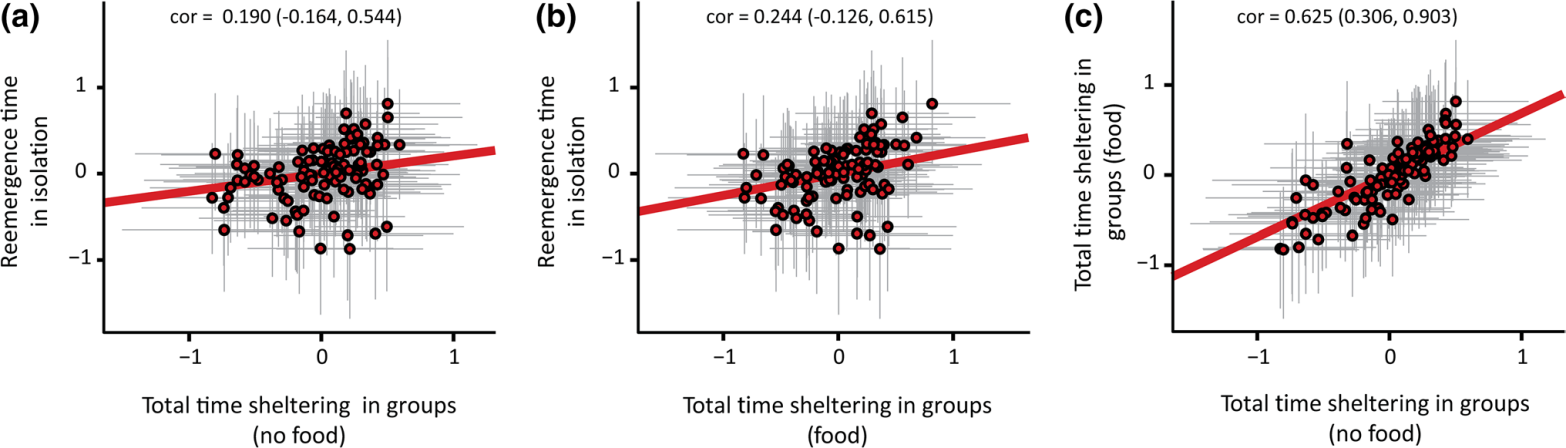
Among-individual correlations between risk-taking behaviour across social and foraging conditions (*n* = 105). Individual plots demonstrate the correlations between individual re-emergence time and shelter use in groups (**a**) without food and (**b**) with food resources available, as well as (**c**) between shelter use in groups with and without food resources. Among-individual correlations ± 89% credible intervals (CrI) are shown for each trait combination, with CrIs that do not overlap zero considered statistically significant. Data represent the best linear unbiased predictor (BLUP) ± 89% CrIs extracted from the Bayesian multivariate model, with trend lines displayed in red

Data were analysed using *R* version 4.0.3 (R Core Team 2019). Two lizards did not complete individual trials and were therefore excluded from analysis. Further, data were excluded from analysis where an individual’s identity could not accurately be obtained during group trials. This resulted in a total of 741 behavioural observations (totalling 387 h) from 105 skinks included in the analysis. We employed a ‘character-state’ approach by using a Bayesian multivariate generalized linear mixed-effects model (*brms* package; Bürkner 2017) to estimate among-individual correlations between each of our behavioural measures. Data were transformed to approximate Gaussian error distributions (see supplementary material). We ran a tri-variate model testing risk-taking behaviour across the different social and foraging conditions. The model contained re-emergence time measured in isolation and total time sheltering in groups with and without food as three separate dependent variables. The model also included population (O’ahu, Kaua’i, Hawai’i), trial number (individual trials = 2; group trials = 3), and SVL as fixed effects, while individual ID (i.e. 1–105) and group ID (i.e. 1–9) were both included as random intercepts. All dependent variables, as well as continuous covariates (i.e. SVL) were scaled (mean = 0, SD = 1) prior to analysis to aid in model fitting. The model was run for a total of 10,000 iterations (1,000 warmup), using four chains, a thinning interval of two, and relatively uninformative, default priors. Model convergence was checked via trace plots, with all Rhat = 1. We report posterior means with 89% credible intervals (CrI) as suggested by McElreath (2020), with inference based on non-overlapping CrIs with zero (see Table S1 for model output). Figures in the main text displaying among-individual correlations (i.e. Figure 2) represent best linear unbiased predictors (i.e. BLUPs) extracted from model predictions (see Fig S1 for plots of raw data). We note, however, that BLUPs were only used for illustrative purposes and that all correlation analyses were performed on the complete dataset using multivariate models, thus allowing an appropriate estimation of uncertainty in the data (Houslay and Wilson 2017).

We used this tri-variate model to estimate the among-individual (i.e. differences between individuals; V_A_), among-group (i.e. differences between groups; V_A-GROUP_), and within individual (i.e. differences within individuals; V_W_) variance for each measure of risk-taking behaviour (i.e. individual re-emergence time, total time sheltering in groups with and without food). These variance estimates were used to calculate the short-term adjusted repeatability (R) of each behaviour. Adjusted repeatability represents the total amount of behavioural variation that is attributable to among-individual differences, after accounting for the variation explained by fixed-effects (Nakagawa and Schielzeth 2010). We also calculated the effect size of the magnitude difference in variance components and repeatability (ΔV_A_, ΔV_A-GROUP_, ΔV_W_, ΔR) between risk-taking behaviour in each context (i.e. in isolation, groups with and without food) to determine how behavioural variation changed across the social and foraging conditions (Royauté and Dochtermann 2021).

In our analysis, we found that individual risk-taking behaviour was not consistent across social conditions (see Results). We, therefore, ran a separate post-hoc analysis to investigate whether this lack of consistency in behaviour across the social conditions was due to the composition of individual personality traits within social groups. More specifically, we investigated whether the boldest, shyest, or median group member’s behavioural phenotype, in individual trials, was a strong predictor of their group member’s behaviour during group trials (thus potentially explaining the lack of individual consistency across the social conditions). To do this, we ran two linear mixed-effects models with total time sheltering when in groups with and without food as two separate dependent variables (*lme4* package; Bates et al. 2015). Individual mean re-emergence scores of the boldest, shyest, and median group member as estimated from individual trials were included as fixed effects while individual ID and group ID were included as random intercepts (see supplementary material). However, the inclusion of group ID resulted in singular model fits, and therefore, group ID was excluded from the final models. For both models, type II Wald tests using Kenward-Roger approximations for denominator degrees of freedom were used to calculate the *P*-values of fixed-effects.

## Results

### Repeatability and variance estimates

Re-emergence time was moderately repeatable when measured in isolation (Table 1). Similarly, total time spent sheltering was also repeatable when measured in groups with and without food resources (Table 1). There were no differences in repeatability, among-individual variation, or within-individual variation in risk-taking behaviour between the contexts (i.e. measured in isolation, in groups with or without food; Table S2). Further, while there was a marginal increase in among-group variance during trials where food was available, there was substantial uncertainty around this estimate with CrIs including zero (Table S2).

**Table 1:** Variance components and adjusted repeatability (± 89% credible intervals) for individual re-emergence time when tested in social isolation (Individual), as well as total time sheltering in groups with and without food resources available

Context	Among–individual (V_A_)	Within–individual (V_W_)	Among–group (V_A-GROUP_)	Repeatability (R)
Individual	0.284 (0.095, 0.471)	0.716 (0.534, 0.883)	—	0.281 (0.118, 0.450)
Group (no food)	0.245 (0.112, 0.369)	0.725 (0.606, 0.846)	0.025 (0, 0.056)	0.245 (0.127, 0.354)
Group (food)	0.276 (0.120, 0.433)	0.679 (0.538, 0.812)	0.096 (0, 0.212)	0.264 (0.123, 0.403)

### Cross-context behavioural correlations

We found no clear evidence for among-individual correlations between re-emergence time when measured in isolation (i.e. when tested individually) and shelter use when measured in groups, with or without food (in the presence of food: cor [89% CrI] = 0.244 [−  0.126, 0.615] Fig. 2b; in the absence of food: cor = 0.190 [−  0.164, 0.544] Fig. 2a; Fig S1a,b), indicating that bolder skinks during individual trials were not also bolder when measured in groups. Because correlations between individual risk-taking behaviour in isolation and in groups (both with and without food) overlapped with zero and were substantially less than 1, this suggests that individuals differed in how they altered their risk-taking behaviour in response to the presence of conspecifics, resulting in a rank-order change of behavioural types across the social conditions (Brommer 2013; Mitchell and Houslay 2021). In contrast, there was a substantial among-individual correlation between shelter use in groups across the foraging conditions (cor = 0.625 [0.306, 0.903]; Fig. 2c; Fig S1c), with bolder individuals during groups trials without food also displaying high boldness when measured in groups with food resources available. This significantly positive correlation between shelter use in groups both with and without food suggest that individual differences in plasticity were minimal, with individuals being largely consistent in their behaviour when measured in groups across the resource conditions.

### Group analysis

**Fig. 3:**
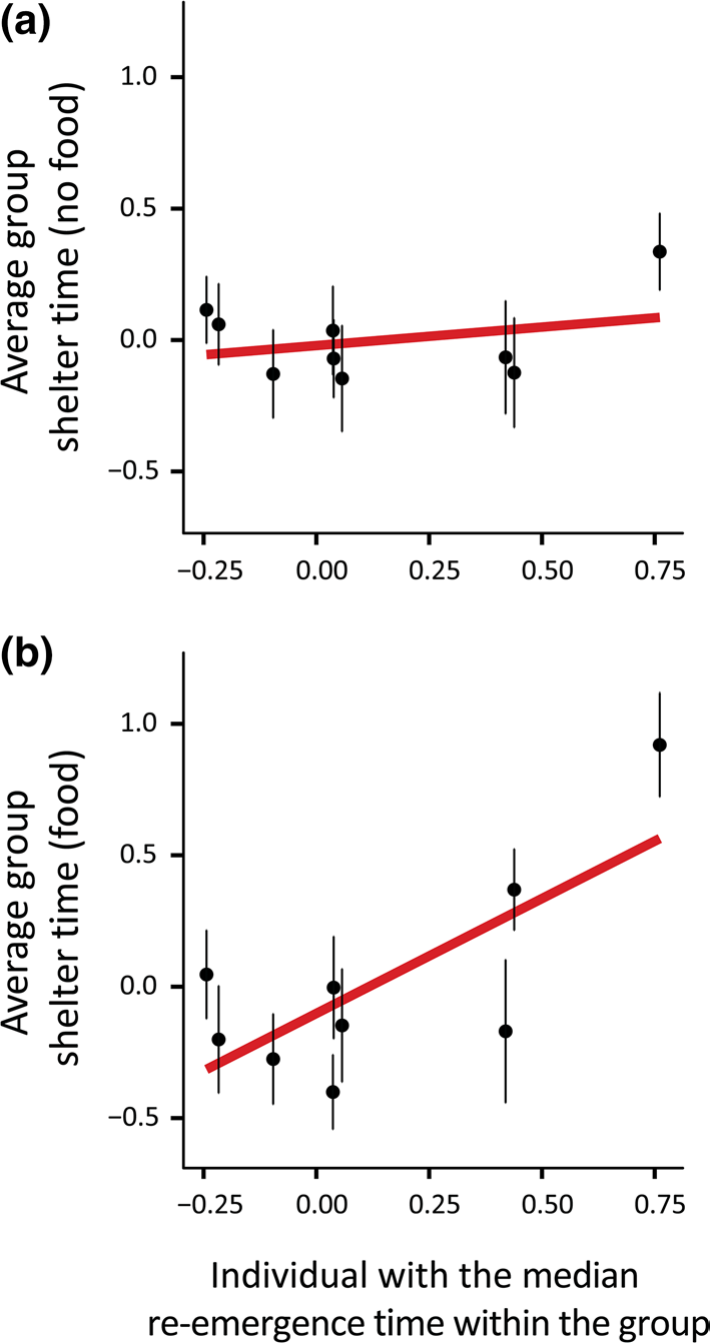
Relationship between the median re-emergence score among the group members tested individually and the average (± standard error) sheltering time during the group trials **a** without food and **b** with food (*n* = 105 skinks in 9 groups). All behavioural scores are presented in transformed and standardized units

Post-hoc analysis found that behavioural types measured during individual trials (i.e. non-social conditions) had a significant effect on the behaviour of conspecifics during social trials; however, this was only true in the foraging context. Specifically, individual sheltering time in groups with food significantly increased with increasing re-emergence times of the median group member, as determined from individual trials (i.e. median re-emergence time; F_1,97_ = 10.51, *P* = 0.002; Fig. 3b; Table S4). In other words, lizard behaviour when in groups with food available was correlated with that of the group member with the median risk-taking score from individual trials. In contrast, there was no effect of either the shyest (i.e. maximum re-emergence time; F_1,87_ = 2.35, *P* = 0.129; Table S4) or boldest (i.e. minimum re-emergence time; F_1,94_ = 2.07, *P* = 0.153; Table S4) individual group member on the total time lizards spent sheltering while in groups with food. Similarly, we found that individual re-emergence scores of the boldest (F_1,99_ = 0.85, *P* = 0.360), shyest (F_1,101_ = 0.45, *P* = 0.506), and median (F_1,99_ = 0.36, *P* = 0.549; Fig. 3a) individual group members had no effect on total time sheltering when in groups without food resources (Table S3).

## Discussion

We demonstrate the importance of the social environment in mediating individual behavioural types. Specifically, we repeatedly measured risk-taking behaviour of individuals in isolation from conspecifics and as part of a social group both in the presence and absence of food resources. We found that risk-taking behaviour was repeatable within each social and foraging condition, but that an individual’s boldness in social isolation did not predict its boldness when in groups. These results highlight that the social environment plays a key role in shaping behavioural traits and suggest that animal behaviour when measured in isolation may not be reflective of individual behavioural types in group settings, particularly in social species.

The finding that individuals which were bolder when measured in isolation from conspecifics were not also bolder when in social groups was surprising, and contrary to predictions. Indeed, these findings are in contrast to prior work in fish, where individuals who were bolder (Magnhagen and Bunnefeld 2009) or faster-moving (Jolles et al. 2017) during trials in isolation, displayed similar behaviours when in groups. However, the current results are in accordance with previous research in cockroaches (*Blaberus discoidalis*), which found that individual differences in light-avoidance behaviour were not maintained across solitary and group contexts (Crall et al. 2016). Together, these results suggest that the influence of the social environment on behaviour may be taxon specific—likely dependent upon the social system of the focal species (Webster and Ward 2011).

Importantly, the lack of correlation between risk-taking behaviour during individual and group trials in the present study was not due to a reduction in among-individual variation when in social groups. In fact, we found similar levels of variation between individuals in their risk-taking behaviour when measured alone, and in groups both with and without food resources. Together, this suggests that individuals may have responded differently to the presence of conspecifics (i.e. social plasticity), resulting in the rank-order of individual behavioural types changing from the non-social to social trials. Previous research in water striders (*Aquarius remiges*) has similarly found individual differences in social plasticity, whereby males differed in how they adjusted their activity in response to increased conspecific density (Montiglio et al. 2017). Further, recent research in delicate skinks also reported that behavioural responses to increasing group sizes may vary among individuals and suggested that this may depend upon an individual’s behavioural type (Littlewood et al. 2021). More specifically, the authors found that while shyer lizards became bolder with increasing group size, the opposite was true for bold lizards (Littlewood et al. 2021). However, this pattern of personality dependent social plasticity cannot entirely explain the current results. For example, if bolder individuals became consistently shyer and shyer individuals became bolder, we would expect to see either a reduction in among-individual variance when in groups (i.e. individuals become more similar to each other) and/or a negative among-individual correlation between risk-taking behaviour during individual and groups trials. Instead, the current research found neither, suggesting that the direction of behavioural change across the social contexts is not well explained by an individuals’ behavioural type. The mechanisms that determine the direction and degree of social behavioural plasticity are not well known. Indeed, whether these individual differences in social plasticity are themselves repeatable and stable over the lifetime of an individual will be an interesting avenue for future studies. Regardless, this research highlights the strong effect of the social environment in shaping behavioural traits.

It should also be noted that differences in assay protocol may partly explain the lack of correlation between risk-taking behaviour during individual and group trials. Unfortunately, logistical constraints required different measures of boldness to be taken in the non-social (i.e. re-emergence time from shelter) and social (i.e. total time in shelter) contexts. Despite both shelter re-emergence latency and total time in the shelter being commonly used measures of boldness (see Methods) and equally repeatable in the current study, the use of different assays may have contributed to the lack of correlation between risk-taking behaviour during individual and group trials. While prior research has found associations between shelter re-emergence and other measures of boldness (Burns 2008; Boulton et al. 2014; Sakai 2020; Ortiz-Jimenez et al. 2022), similar studies have reported contrasting results (Beckmann and Biro 2013; Yuen et al. 2017; see Carter et al. 2013 for a broader discussion). Thus, where possible, future experiments should measure the same behavioural traits in both non-social and social contexts. Moreover, repeatedly testing the same behavioural traits across varying densities of conspecifics (e.g. Rieucau et al. 2010; Littlewood et al. 2021) will also be a valuable avenue for further research to confirm the role of the social environment in mediating individual behavioural types.

Further, we found tentative evidence that individual behaviour when in social groups was affected by the behavioural types of key group members. Specifically, individual risk-taking behaviour when in groups was affected by the behavioural type of the median individual group member (as determined from individual trials). However, we advise some caution in interpreting this result given the limited number of social groups used in the current study and because the relationship appears to be driven by the behaviour of a few groups. Nevertheless, previous studies have similarly shown that the behaviour of conspecifics can influence individual behavioural types (Guayasamin et al. 2017; King et al. 2015; Fürtbauer and Fry 2018; Munson et al. 2021; Kelly et al. 2020). For example, the social tendencies of three-spined sticklebacks changed after being housed within stable social groups, whereby group social tendencies were driven by the most social individual within each tank (Munson et al. 2021). Intriguingly, however, we report that the effect of an individual’s behavioural type—as determined from individual trials—on group member behaviour during social trials was observed despite finding no correlation between an individual’s behaviour across the non-social and social conditions. This suggests that some component of an individual’s behavioural type, as measured during individual trials, may be expressed in the presence of conspecifics, even when the behavioural type of that individual is not conserved across social conditions. How and why this occurs is not clear but will be an important topic for further research. Nevertheless, these results tentatively suggest that the composition of behavioural types within social groups may alter individual-level behavioural traits. Future experiments which experimentally manipulate group composition in a larger number of groups will be needed to better understand the role of group-member behaviour in mediating individual behavioural types.

Interestingly, we only found an effect of group member behaviour on individual behavioural types during trials where food was available, with the behavioural types of conspecifics having no significant effect on individual behaviour in social groups in the absence of food. While again we advise some caution in interpreting these group-level results, we surmise that this may be due to differences in the salience of social cues between the two contexts. Indeed, previous research has shown that social cues can provide valuable information about resource location (Aplin et al. 2012; Pérez-Cembranos and Pérez-Mellado 2015). Lizards during the current study may therefore have been more attentive to the behaviour of conspecifics during trials where food was available, as this may have provided useful information about resource location. This potential increase in the salience of social cues under high resource conditions could explain why individual boldness was only influenced by the behaviour of conspecifics during trials where food was available. Further research is needed to better understand the context-specific role of the social environment in mediating behaviour. In any case, these results indicate a complex relationship between the social environment and resource availability in shaping individual-level behaviour.

In contrast to the lack of consistency in boldness across social contexts, we found that individual differences in boldness were consistent across situations that differed in an important ecological context: the absence versus presence of food. This is in line with prior research in three-spined sticklebacks which found that individual risk-taking behaviour was consistent across foraging conditions (MacGregor et al. 2021). The general issue of consistency of behavioural types across ecological contexts is important because, among other things, it can generate across-context trade-offs (Sih et al. 2004, 2012). For instance, individuals that are bolder, both in the presence and absence of predators, might have higher feeding rates in the absence of predators, but higher mortality when predators are present, compared to shyer individuals. Previous studies have, indeed, found consistency in risk-taking behaviours in the presence versus absence of predation risk (e.g. Balaban-Feld et al. 2018), and also consistency across foraging, antipredator and mating contexts (e.g. Johnson and Sih 2007). Whether these consistent individual differences in boldness across foraging conditions found in the current study are maintained in the presence of predation risk—where the costs of risky behaviours are most apparent—will be key in understanding the role of trade-offs in mediating consistent individual differences in behaviour.

In summary, we found that an individual’s behaviour when measured in isolation did not predict their behaviour when in a social setting. We contend that this was, in part, driven by the specific composition of personality types within the group, with individual behavioural traits influenced by the behavioural types of key group members—at least under high resource conditions. However, we did find that individual behavioural types were consistent across foraging conditions when in social groups. Collectively, these findings suggest that (i) the social environment is an important mediator of individual behaviour, and (ii) the influence of the social environment on individual behaviour may itself be influenced by the environmental context (i.e. food availability). As individual differences in behaviour have been associated with survival (Ballew et al. 2017; Smith and Blumstein 2010; Moirón et al. 2019) and reproductive success (Schuett et al. 2010; Munson et al. 2020), understanding how changing environmental conditions may shape behavioural variation within populations, and how selection may act on that variation, is an important topic for future research. Nevertheless, the current results highlight the importance of understanding how social conditions contribute to among-individual differences in behaviour.

## Supplementary Information

Below is the link to the electronic supplementary material.

Supplementary file1 (PDF 442 KB)

## Data Availability

The data to reproduce the results reported in this analysis are publicly available at the Open Science Framework online repository (https://osf.io/e6us9/).

## Code availability

The statistical code to reproduce the results reported in this analysis are publicly available at the Open Science Framework online repository (https://osf.io/e6us9/).
