# Supplementary material for: Social context mediates the expression of a personality trait in a gregarious lizard
Source: Oecologia. 2022 Sep 29;200(3-4):359–69. doi: 10.1007/s00442-022-05269-7 (PMC9675666; doi:10.1007/s00442-022-05269-7)
Supplement: Supplementary file 1 — Supplementary file1 (PDF 442 KB) [file 442_2022_5269_MOESM1_ESM.pdf]

Social context mediates the expression of a personality trait in a gregarious lizard

Jack A. Brand[1]*, Annalise C. Naimo[1], Marcus Michelangeli[1,2,3], Jake M. Martin[1,3], Andrew Sih[2,†], Bob B.M. Wong[1,†], David G. Chapple[1,†]

[1]School of Biological Sciences, Monash University, Melbourne, VIC, Australia

[2]Department of Environmental Science and Policy, University of California, Davis, CA, USA

[3]Department of Wildlife, Fish, and Environmental Studies, Swedish University of Agricultural Sciences, Umeå, Sweden

[†]Co-senior authors: These authors contributed equally to this work

Author for correspondence

*Jack A. Brand

Email: jack.brand@monash.edu

Electronic Supplementary Material

METHODS

Skink collection and group trials

Skinks were originally collected from three populations across their invasive range in the Hawaiian Islands. More specifically, lizards were collected from populations on the islands of O'ahu (*n* = 35), Kaua'i (*n* = 36), and Hawai'i (*n* = 36). For group trials, lizards were split into three groups per population with 12 individuals per group. However, one group from the O'ahu population contained 11 skinks (*n* = 35 does not split evenly into three groups). Similarly, two lizards from the Hawai'i population were missing individual data and were therefore excluded from analysis. However, these lizards were still physically present during the group trials. This resulted in a total of 105 lizards included in the final analysis.

Statistical analysis

We estimated among-individual correlations between risk-taking behaviour scored when alone, as well as when in social groups with and without food resources. To meet the assumption of normality, individual re-emergence time was log_{10} transformed, while total time spent sheltering in groups with and without food resources were both square-root transformed. We fitted a Bayesian multivariate generalized linear mixed-effects model containing each of our three behavioural traits included as separate dependent variables. Models included population (O'ahu, Kaua'i, Hawai'i), trial (1-3), and SVL as fixed effects, while individual ID (all models) and group ID (group models only) were included as random intercepts to account for repeated measures. Further, we allowed correlations to vary amongst individuals across the three separate response variables to estimate the among-individual correlations between these three behavioural measures. However, there were some missing values in the dataset. For example, where an individual could not be accurately identified from colour codes during social trials or due to an increased number of repeated measures performed in social settings

(i.e. 3 repeated measures) when compared to individual trials (i.e. 2 repeated measures). Therefore, we used multiple imputation to account for missing values in each response variable as suggested by Nakagawa and Freckleton (2008, 2011). This was done during model fitting using the *mi*() function in the *brms* package (Bürkner 2017). Multiple imputation in the *brms* package infers missing values from the posterior predictive distribution of the relevant response variable. This allowed us to retain the maximum amount of data in the model to estimate among-individual correlations between our three measures of risk-taking behaviour.

Further, linear mixed effects models were used as a post-hoc analysis to investigate whether an individual's behaviour within a social group was influenced by the behavioural types of group members as estimated from individual trials. To do this we used the transformed and scaled (i.e. mean = 0, SD = 1) total time sheltering in groups with and without food resources as two separate dependent variables. Initial models included the transformed and scaled mean individual re-emergence scores across the two trials of the boldest, shyest, and median group member as fixed effects, while individual ID was included as a random intercept to account for repeated measures. The significance of fixed-effects was determined using type II Wald tests with Kenward-Roger approximations for degrees of freedom (*car* package; Fox and Weisberg 2019).

Results

Table S1. Parameter estimates (89 % CrI) of fixed and random effects from Bayesian multivariate generalized linear mixed-effects model output. Credible intervals of fixed effects that do not include zero are considered statistically significant and are indicated in bold. Note: Random effects are given in standard deviations (sd).

	Individual re-emergence	Sheltering in groups (no food)	Sheltering in groups (food)
Fixed effects			
Intercept	−0.20 (−0.55, 0.15)	**−0.69 (−0.99, −0.38)**	0.02 (−0.39, 0.43)
Population			
O’ahu	0.22 (−0.08, 0.53)	0.08 (−0.25, 0.42)	**0.55 (0.06, 1.04)**
Hawai’i	−0.29 (−0.59, 0.02)	0.07 (−0.26, 0.40)	0.03 (−0.45, 0.50)
Trial	0.15 (−0.04, 0.34)	**0.32 (0.22, 0.41)**	−0.09 (−0.20, 0.02)
Length	−0.02 (−0.14, 0.11)	−0.04 (−0.15, 0.08)	−0.02 (−0.15, 0.10)
Random effects			
sd (Individual identity)	0.52 (0.31, 0.69)	0.49 (0.35, 0.62)	0.52 (0.36, 0.67)
sd (Group identity)	NA	0.12 (0.01, 0.30)	0.25 (0.03, 0.57)
Residual	0.84 (0.75, 0.96)	0.85 (0.78, 0.92)	0.82 (0.74, 0.91)

Table S2. Effect size (± 89 % CrI) for the magnitude difference in variance components and repeatability (ΔV_A, ΔV_W, $\Delta V_{A\text{-}GROUP}$, ΔR) between risk-taking behaviour in each context (i.e. in isolation, groups with and without food).

**Contrast**	**ΔV_A**	**ΔV_W**	**$\Delta V_{A\text{-}GROUP}$**	**ΔR**
Isolation – group (no food)	0.038 (−0.195, 0.260)	−0.009 (−0.236, 0.197)	—	0.036 (−0.161, 0.239)
isolation – group (food)	0.007 (−0.239, 0.248)	0.037 (−0.197, 0.255)	—	0.017 (−0.198, 0.232)
Group (no food) – Group (food)	−0.031 (−0.227, 0.175)	0.046 (−0.134, 0.238)	−0.071 (−0.24, 0.08)	−0.019 (−0.198, 0.158)

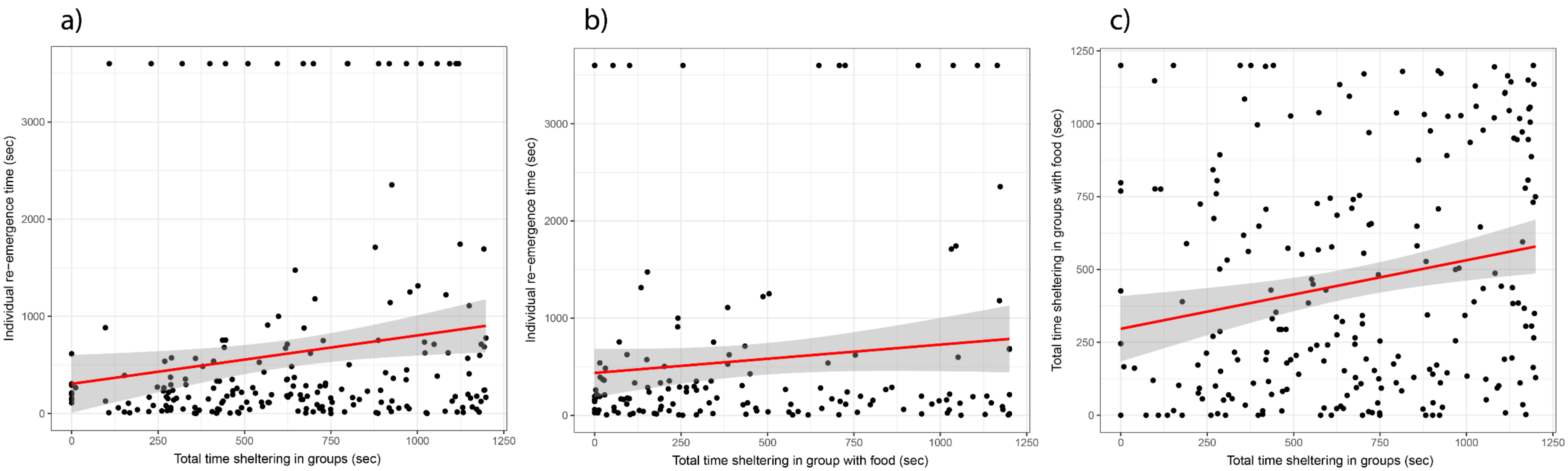

Fig S1. Plots of the raw, untransformed data showing correlations between (a) individual re-emergence times in isolation and total time sheltering in groups without food, (b) individual re-emergence times in isolation and total time sheltering in groups with food, and (c) total time sheltering in groups both with and without food. Trend lines are displayed in red with shaded areas denoting standard error.

Table S3. Output from linear mixed-effects model investigating the influence of the shyest, boldest, and median individual (as estimated from re-emergence times during individual trial) on the total time sheltering in group trials without food present. Individual ID was included as a random effect to account for repeated measures.

**Response**	**Predictors**		
Total time sheltering within groups (no food)	**Fixed effects**	***F***	***P***
	Shyest group member (i.e. individual with maximum re-emergence time within the group)	0.45	0.506
	Boldest group member (i.e. individual with minimum re-emergence time within the group)	0.85	0.360
	Median group member (i.e. individual with the median re-emergence time within the group)	0.36	0.549
	**Random effects**	**var**	
	ID	0.216	
	Residual	0.799	

Table S4. Output from linear mixed-effects model investigating the influence of the shyest, boldest, and median individual (as estimated from re-emergence times during individual trial) on the total time sheltering in group trials with food present. Individual ID was included as a random effect to account for repeated measures. Significant *P* values are indicated in bold.

**Response**	**Predictors**		
Total time sheltering within groups (food)	**Fixed effects**	***F***	***P***
	Shyest group member (i.e. individual with maximum re-emergence time within the group)	2.35	0.129
	Boldest group member (i.e. individual with minimum re-emergence time within the group)	2.07	0.153
	Median group member (i.e. individual with the median re-emergence time within the group)	10.51	**0.002**
	**Random effects**	**var**	
	ID	0.252	
	Residual	0.661	

REFERENCES

Bürkner PC (2017) brms: an R package for Bayesian multilevel models using Stan. J Stat Softw 80:1–28. doi:10.18637/jss.v080.i01.

Fox J, Weisberg S (2019) An R Companion to Applied Regression. Third. Thousand Oaks, CA: Sage.

Nakagawa S, Freckleton RP (2008) Missing inaction: the dangers of ignoring missing data. Trends Ecol Evol 23:592–596. doi:10.1016/j.tree.2008.06.014.

Nakagawa S, Freckleton RP (2011) Model averaging, missing data and multiple imputation: a case study for behavioural ecology. Behav Ecol Sociobiol 65:103–116. doi:10.1007/s00265-010-1044-7.
